# Supplementary material for: Cerebral microcirculation mapped by echo particle tracking velocimetry quantifies the intracranial pressure and detects ischemia
Source: Nat Commun. 2022 Feb 3;13:666. doi: 10.1038/s41467-022-28298-5 (PMC8814032; doi:10.1038/s41467-022-28298-5)
Supplement: Supplementary file 1 — Supplementary Information [file 41467_2022_28298_MOESM1_ESM.pdf]

Supplemental Information

Cerebral Microcirculation Mapped by Echo Particle Tracking Velocimetry Quantifies the Intracranial Pressure and Detects Ischemia

Authors: Zeng Zhang[1†], Misun Hwang[2,3†], Todd J. Kilbaugh[4], Anush Sridharan[2], Joseph Katz[1]*

Affiliations:

[1]Department of Mechanical Engineering, Johns Hopkins University, Baltimore, Maryland, USA.

[2]Department of Radiology, Children's Hospital of Philadelphia, Philadelphia, Pennsylvania, USA.

[3]Department of Radiology, Perelman School of Medicine, University of Pennsylvania, Philadelphia, Pennsylvania, USA.

[4]Department of Anesthesiology and Critical Care Medicine, Children's Hospital of Philadelphia, Philadelphia, Pennsylvania, USA.

* Corresponding author: katz@jhu.edu

† Zeng Zhang and Misun Hwang contribute equally to the present work.

Supplemental Methods

Time-intensity based dynamic CEUS analysis

To perform the time-intensity curve based dynamic CEUS analysis, we used both disruption & replenish as well as bolus injection sequences. Several parameters, namely the peak intensity, rise time, average wash-in/refilling slope, area under the curve of the wash-in/refilling phase (WIAUC), perfusion index (WIAUC/rise time), refilling rate (disruption-replenish sequences only), and the max intensity & wash-in/refilling slope ratio, were analyzed following procedures described previously [1,2]. The analysis was performed based on data recorded in the cortex, thalamus, and the whole brain, where the selection of these regions also followed previous procedures[1]. Due to the uncertainty in identifying the exact time point where the data first deviated from zero for the bolus injection or plateaus for the disruption & replenish sequence (1-2 s, as much as 25-50% of the rise time), the comparison between wash-in/refilling curves was based on the time that the intensity increased from 25% to 75% of the maximum value.

Vesselness filter

The selection of cortical regions of interest and exclusion of the big blood vessels were performed both manually and by implementing an automated procedure. The latter was aimed at assuring that the empirical relations between CMC_{cort} and ICP were not biased by/sensitive to the selection of micro-vascular regions. This approach involved several steps. First, the entire cortical region was roughly defined/identified manually, including the large vessels (highlighted area in step 1 of Fig. S1b). Then a vesselness filter [3], which was commonly used in angiography to enhance the blood vessels, was used for identifying the big vessels. This method convolved the input image with gaussian filters of several scales (sigma=4-8 at the present magnification) and calculated the eigenvalues of the local normalized Hessians. A vesselness function, whose exact form could be found in Jerman et al. [3], combined the eigenvalues at all scales in each pixel in a way that enhanced tubular structures and suppressed others. Prior to filtering, the trajectory heatmap was normalized by its maximum value to obtain a pixel value range of 0 to 1. Subject to the selected filter scales, this procedure enhanced blood vessels larger than 400μm while suppressing the signals of smaller ones, as demonstrated by the filtered image in the step 2 of Fig. S1b. Regions with vesselness values higher than 0.25 were binarized and dilated by two pixels in all directions (step 3 of Fig. S1b) to ensure that all the regions belonging to the major vessels were covered. These regions were removed from the roughly selected cortical area (step 4 of Fig. S1b). Remaining isolated regions smaller than 50 pixels were also removed. The resulting area consisted of several subregions containing vessels smaller than 400μm (highlighted regions in the step 4 of Fig. S1b). The region selection was performed using the baseline data for each pig, where the vessels were

expected to have the largest width, and then used for all ICP levels. All the image processing was performed using the MATLAB Image Processing Toolbox.

A comparison between the results obtained for the manually selected regions and those determined using the vesselness filtering are presented in Fig. S1c-e. Results are shown separately for the original (solid symbol) and validation (hollow symbol) cohorts, as well as for regions 2+3+4 (black), and regions A+2+3+4 (red). Clearly, the values of CMC_{cort} obtain for regions 2-4 defined by manual and vesselness filtering are the same (Fig. S1c), with a linear correlation coefficient exceeding 0.98 (0.99 for the original cohort). Bland-Altman analyses for regions 2-4 show that the average differences between results are 0.03mm/s for the original data and 0.02mm/s for the validation cohort, (Fig. S1d and e, respectively), with a 95% confidence interval about ±0.2mm/s for both datasets. Extending the microvascular area to include A does not affect the result significantly.

Supplemental Figures

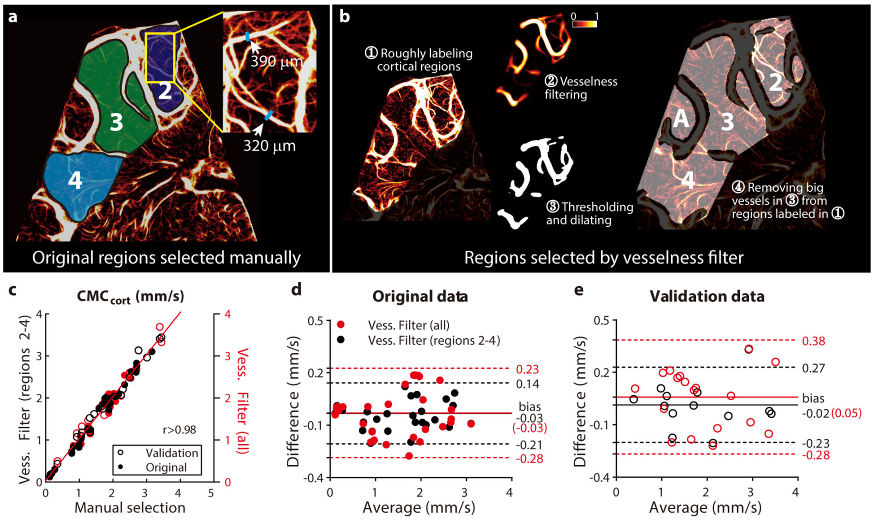

Figure S1 Comparison of the CMC results calculated based on the regions selected manually and by vesselness filter. (**a**) The cortical regions of interest selected manually. The insert shows a magnified image of the largest involved blood vessel whose diameter is about 390μm. (**b**) Steps for removing the major vessels in the cortical regions of interest by the vesselness filter. (**c**) Scatter plot of the CMC_{cort} results for the original and validation data based on the regions selected manually and by vesselness filter. (**d**, **e**) The Bland-Altman analysis of the CMC_{cort} results acquired based on the regions selected manually and by vesselness filter. (d) original data, (e) validation data. For (c-e), the original data is marked by solid symbols while the validation cohort by hollow symbols. Red markers indicate results obtained based on vesselness filtered regions A+2+3+4, while black ones show the result obtained based on vesselness filtered regions 2+3+4. Source data are provided as a Source Data file.

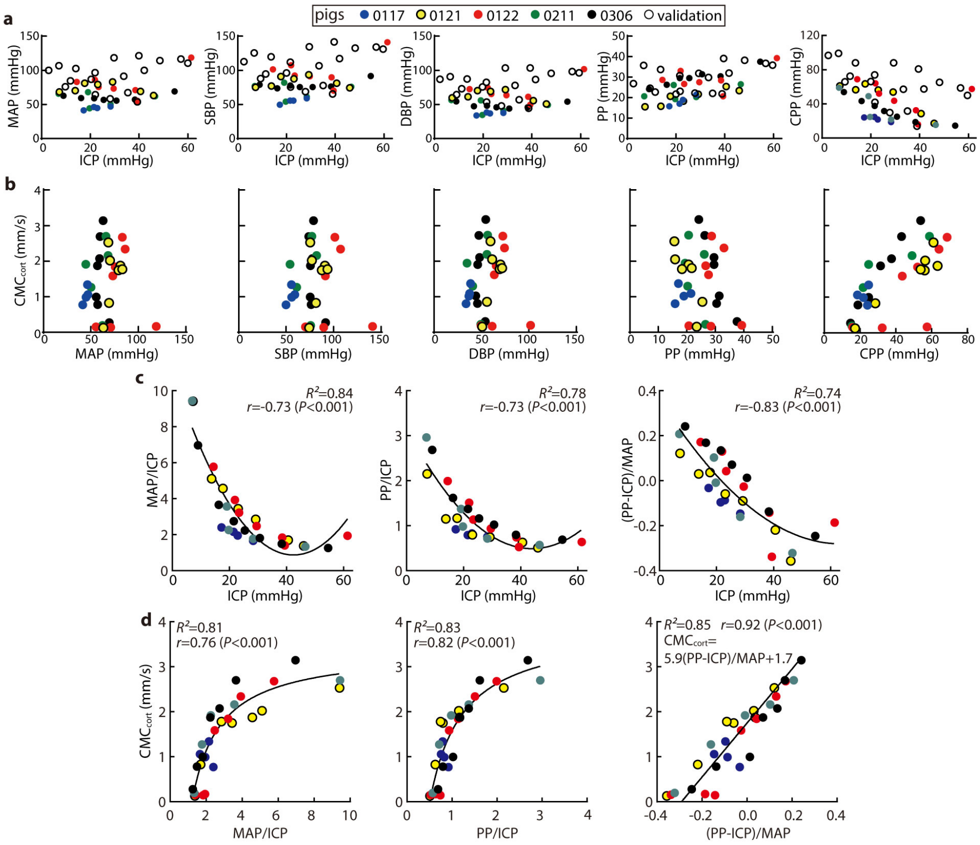

Figure S2 Exploring different combinations of perfusion and hemodynamic parameters aimed at improving the correlation with the ICP. (**a**) Relationships between hemodynamic parameters and ICP for all the piglets. From left to right: MAP, systolic (SBP) and diastolic blood pressure (DBP), pulse pressure (PP=SBP-DBP), and cerebral perfusion pressure (CPP=MAP-ICP). (**b**) Variations of the CMC_{cort} with hemodynamic parameters. From left to right: MAP, SBP, DBP, PP, and CPP. (**c**) Variations of combinations of hemodynamic parameters with ICP, including MAP/ICP , PP/ICP (PP-ICP)/MAP . (**d**) Variations of CMC_{cort} with combinations of hemodynamic parameters, including MAP/ICP , PP/ICP (PP-ICP)/MAP . For (c, d), each plot contains a least square fitted curve along with its R^2, as well as *r* and *P* for the two-tailed Pearson correlation. Source data are provided as a Source Data file.

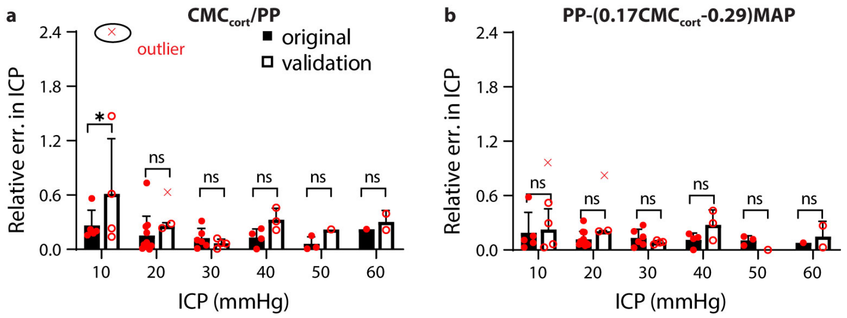

Figure S3 The relative error in ICP prediction. (**a**) The relative errors in ICP prediction based on the parabolic function in Fig. 3c for the original (closed bar) and validation (open bar) data. (**b**) The relative errors in ICP prediction based on the linear function in Fig. 3j for the original and validation data. For (a, b), the dot plots are provided with the bar plots, where solid dots represent original data, hollow ones represent the validation data, and crosses represent outliers. In both bar plots, the bar height denotes the mean value with the error bar showing the standard deviation. For ICP groups of 10, 20, 30, 40, 50, and 60mmHg, there are n=5, 11, 6, 4, 3, and 1 independent cases respectively for the original data, and n=4, 2, 4, 3, 1, and 2 for the validation data. Here, two-way ANOVA multiple comparison results with Benjamini-Hochberg correction are also demonstrated, where 'ns' P>0.05, '*' P<0.05, '**' P<0.01, and '***' P<0.001. Specifically, in (a), the P values are 0.032, 0.556, 0.706, 0.273, 0.557, and 0.775 for increasing ICP levels; in (b), the P values are 0.682, 0.377, 0.564, 0.103, 0.493, and 0.675 respectively. Source data are provided as a Source Data file.

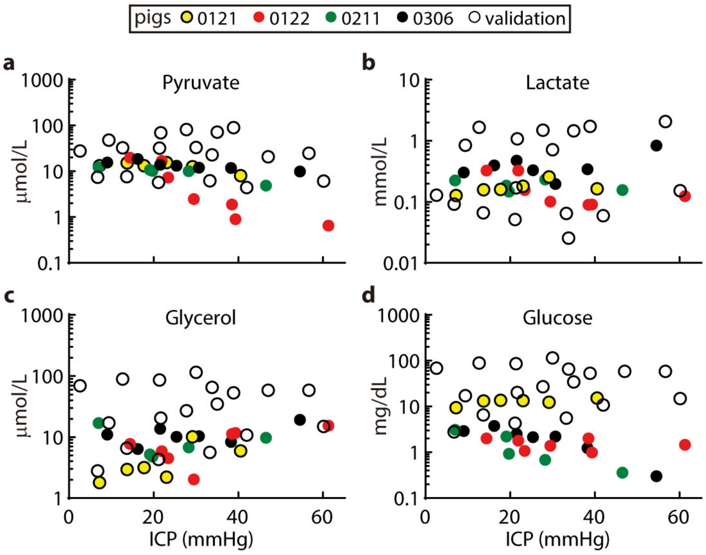

Figure S4 Microdialysis results. (**a**) Pyruvate. (**b**) Lactate. (**c**) Glycerol. (**d**) Glucose. Source data are provided as a Source Data file.

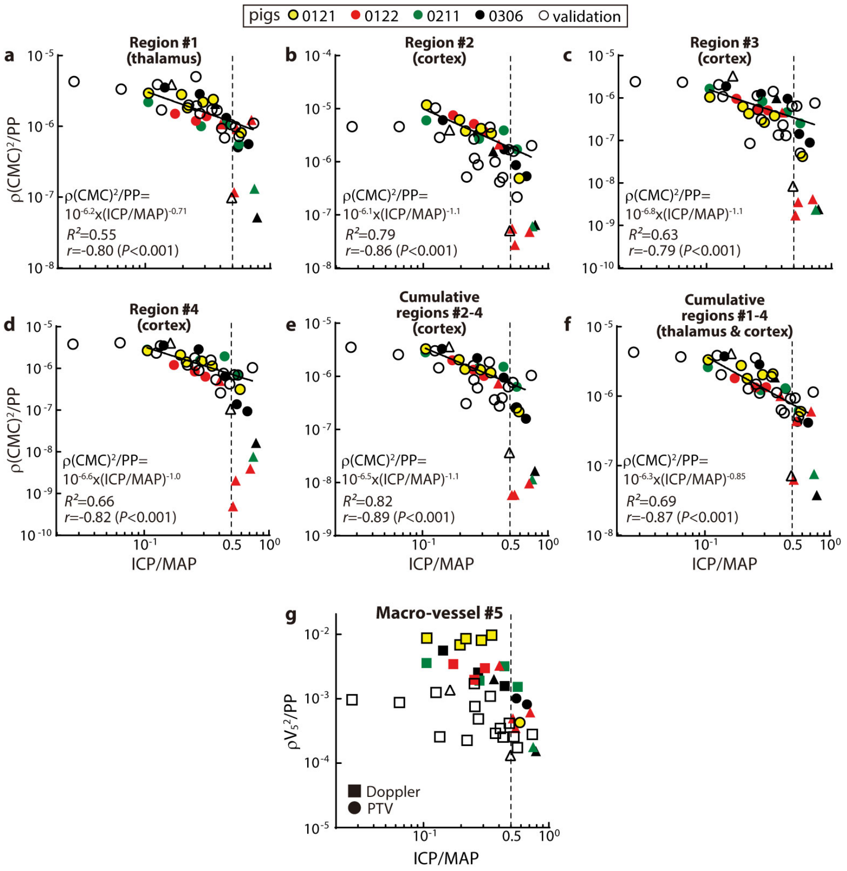

Figure S5 Relationships between nondimensional perfusion parameters and ICP/MAP in different parts of the brain. The locations of measurements include microcirculation in the: (**a**) Thalamus, and cortical regions #2 (**b**), 3 (**c**), and 4 (**d**), as well as: (**e**) the combined cortical microcirculation in regions #2-4, (**f**) The combined microcirculation in regions #1-4, and (**g**) the velocity in macro blood vessel #5. In (a-g), the colored symbols represent the original data while the hollow ones show the validation data. The cases whose microdialysis data are not available are not presented. All cases whose L/P>30 are marked in triangle. In (a-f), the fitted functions are based on the data of the original cohort, along with their R^2, the correlation coefficients and P values for the two-tailed Pearson correlation. Source data are provided as a Source Data file.

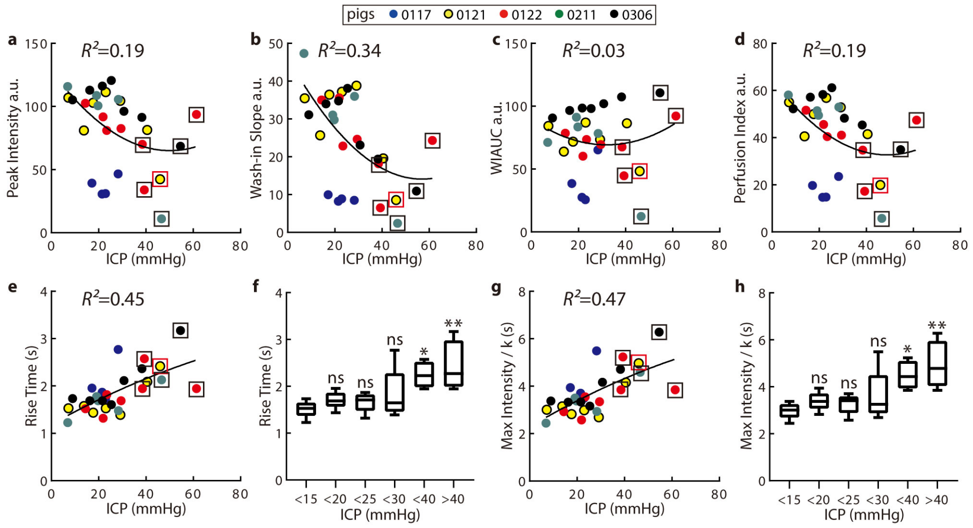

Figure S6 The time-intensity curve analysis of bolus injection sequences in the cortex. (**a**) Peak intensity. (**b**) Average wash-in slope (k). (**c**) Area under the curve of the wash-in phase (WIAUC). (**d**) Perfusion Index: WIAUC/rise time. (**e**) Rise time. (**f**) The statistical differences of the rise time for different ICP levels. (**g**) Max intensity/k. (**h**) The statistical differences of max-intensity/k for different ICP levels. The ischemia cases are marked by the black boxes. The microdialysis result for the case in red box is not available. The R^2 for each parabolic fit is provided in the top left of (a-e, g). For f and h, the whiskers show minima and maxima, the box extends from the 25th to 75th percentiles, and the bar in the middle indicates the median. There are n=5, 5, 6, 6, 4, and 4 independent cases for each increasing ICP level. Moreover, one-way ANOVA multiple comparison results with Dunnett's correction are also demonstrated, where 'ns' P>0.05, '*' P<0.05, and '**' P<0.01. Specifically, in (f), the P values are 0.854, 0.941, 0.423, 0.021, and 0.004 for the increasing ICP level; in (h), the P values are 0.820, 0.935, 0.416, 0.016, and 0.002 respectively. Source data are provided as a Source Data file.

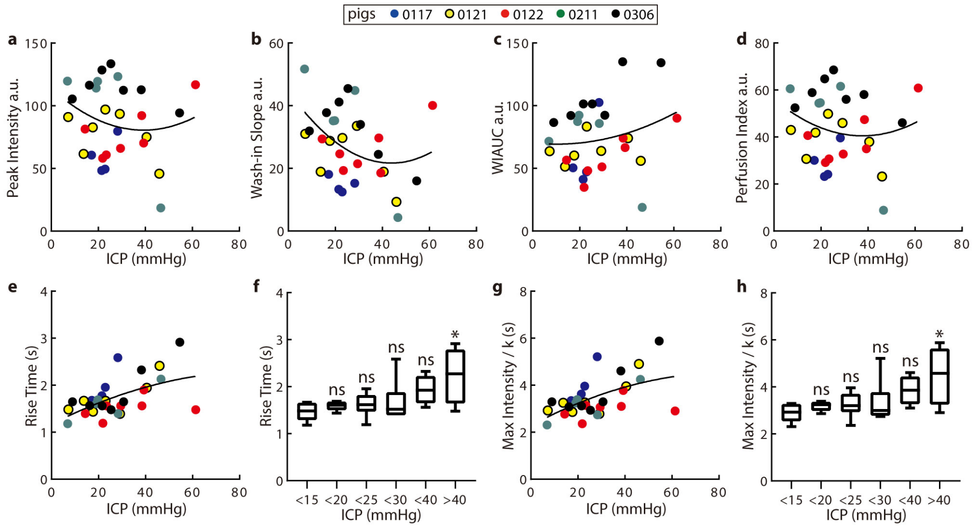

Figure S7 The time-intensity curve analysis of bolus injection sequences in the thalamus. (**a**) Peak intensity. (**b**) Average wash-in slope (k). (**c**) Area under the curve of the wash-in phase (WIAUC). (**d**) Perfusion Index: WIAUC/rise time. (**e**) Rise time. (**f**) The statistical differences of the rise time for different ICP levels. (**g**) Max intensity/k. (**h**) The statistical differences of max-intensity/k for different ICP levels. For f and h, the whiskers show minima and maxima, the box extends from the 25th to 75th percentiles, and the bar in the middle indicates the median. There are n=5, 5, 6, 6, 4, and 4 independent cases for each increasing ICP level. Moreover, one-way ANOVA multiple comparison results with Dunnett's correction are also demonstrated, where 'ns' P>0.05, '*' P<0.05, and '**' P<0.01. Specifically, in (f), the P values are 0.975, 0.938, 0.807, 0.218, and 0.015 for the increasing ICP level; in (h), the P values are 0.962, 0.904, 0.790, 0.216, and 0.014 respectively. Source data are provided as a Source Data file.

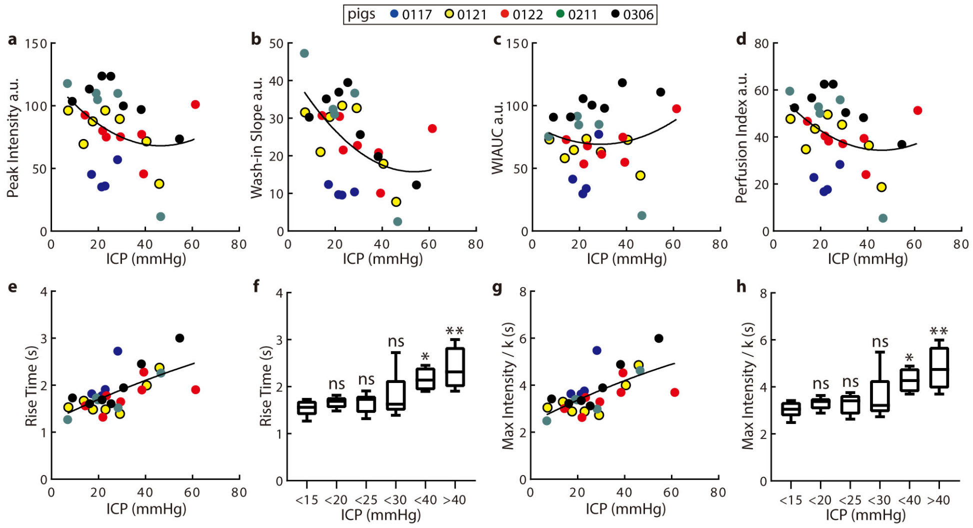

Figure S8 The time-intensity curve analysis of bolus injection sequences in the whole coronal plane. (**a**) Peak intensity. (**b**) Average wash-in slope (k). (**c**) Area under the curve of the wash-in phase (WIAUC). (**d**) Perfusion Index: WIAUC/rise time. (**e**) Rise time. (**f**) The statistical differences of the rise time for different ICP levels. (**g**) Max intensity/k. (**h**) The statistical differences of max-intensity/k for different ICP levels. For f and h, the whiskers show minima and maxima, the box extends from the 25th to 75th percentiles, and the bar in the middle indicates the median. There are n=5, 5, 6, 6, 4, and 4 independent cases for each increasing ICP level. Moreover, one-way ANOVA multiple comparison results with Dunnett's correction are also demonstrated, where 'ns' P>0.05, '*' P<0.05, and '**' P<0.01. Specifically, in (f), the P values are 0.971, 0.972, 0.564, 0.037, and 0.003 for the increasing ICP level; in (h), the P values are 0.960, 0.965, 0.544, 0.041, and 0.003 respectively. Source data are provided as a Source Data file.

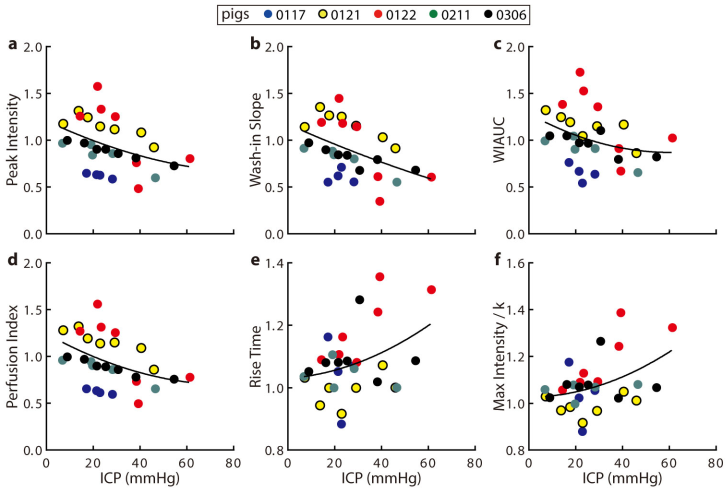

Figure S9 The time-intensity curve analysis of bolus injection sequences for the values of cortex/thalamus. (**a**) Peak intensity. (**b**) Average wash-in slope (k). (**c**) Area under the curve of the wash-in phase (WIAUC). (**d**) Perfusion Index: WIAUC/rise time. (**e**) Rise time. (**f**) Max intensity/k. Source data are provided as a Source Data file.

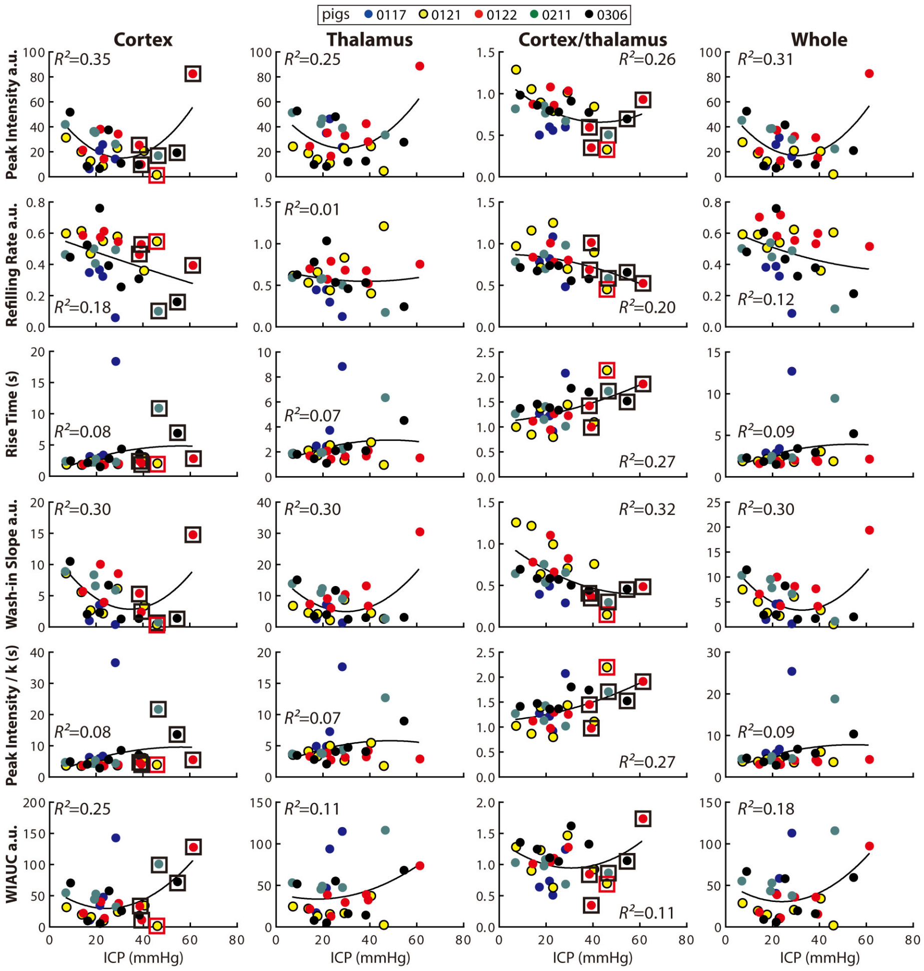

Figure S10 The time-intensity curve analysis of disruption & replenish sequences. Columns (left to right): values of cortex, thalamus, cortex/thalamus, and the whole coronal plane. Rows (top to bottom): parameters including peak intensity, refilling rate, rise time, average wash-in slope (k), max intensity/k, and area under the curve of the wash-in phase (WIAUC). The ischemia cases are marked by the black boxes. The microdialysis result for the case in red box is not available. The R^2 for each parabolic fit is provided. Source data are provided as a Source Data file.

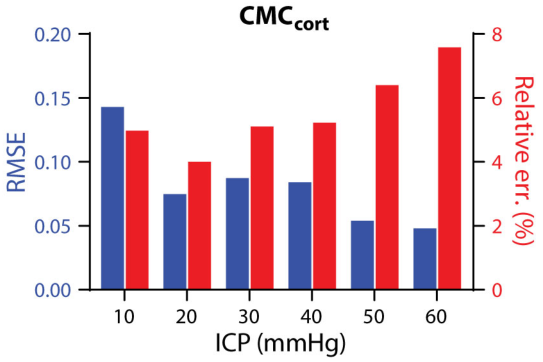

Figure S11 Variations of the uncertainty in CMC_{cort} measurement for different ICP levels. Source data are provided as a Source Data file.

Supplemental References

1. Hwang, M. *et al.* Novel Quantitative Contrast-Enhanced Ultrasound Detection of Hypoxic Ischemic Injury in Neonates and Infants: Pilot Study 1. *J. Ultrasound Med.* **38**, 2025–2038 (2019).
2. Greis, C. Quantitative evaluation of microvascular blood flow by contrast-enhanced ultrasound (CEUS). *Clin. Hemorheol. Microcirc.* **49**, 137–149 (2011).
3. Jerman, T., Pernuš, F., Likar, B. & Špiclin, Ž. Enhancement of vascular structures in 3D and 2D angiographic images. *IEEE Trans. Med. Imaging* **35**, 2107–2118 (2016).
