# Supplementary material for: Cerebral microcirculation mapped by echo particle tracking velocimetry quantifies the intracranial pressure and detects ischemia
Source: Nat Commun. 2022 Feb 3;13:666. doi: 10.1038/s41467-022-28298-5 (PMC8814032; doi:10.1038/s41467-022-28298-5)
Supplement: Supplementary file 3 — Reporting Summary [file 41467_2022_28298_MOESM3_ESM.pdf]

nature portfolio

Corresponding author(s): Joseph Katz

Last updated by author(s): Dec 18, 2021

Reporting Summary

Nature Portfolio wishes to improve the reproducibility of the work that we publish. This form provides structure for consistency and transparency in reporting. For further information on Nature Portfolio policies, see our Editorial Policies and the Editorial Policy Checklist.

Statistics

For all statistical analyses, confirm that the following items are present in the figure legend, table legend, main text, or Methods section.

n/a	Confirmed	
☐	☒	The exact sample size (*n*) for each experimental group/condition, given as a discrete number and unit of measurement
☐	☒	A statement on whether measurements were taken from distinct samples or whether the same sample was measured repeatedly
☐	☒	The statistical test(s) used AND whether they are one- or two-sided<br>*Only common tests should be described solely by name; describe more complex techniques in the Methods section.*
☐	☒	A description of all covariates tested
☐	☒	A description of any assumptions or corrections, such as tests of normality and adjustment for multiple comparisons
☐	☒	A full description of the statistical parameters including central tendency (e.g. means) or other basic estimates (e.g. regression coefficient) AND variation (e.g. standard deviation) or associated estimates of uncertainty (e.g. confidence intervals)
☐	☒	For null hypothesis testing, the test statistic (e.g. *F*, *t*, *r*) with confidence intervals, effect sizes, degrees of freedom and *P* value noted<br>*Give P values as exact values whenever suitable.*
☒	☐	For Bayesian analysis, information on the choice of priors and Markov chain Monte Carlo settings
☒	☐	For hierarchical and complex designs, identification of the appropriate level for tests and full reporting of outcomes
☐	☒	Estimates of effect sizes (e.g. Cohen's *d*, Pearson's *r*), indicating how they were calculated

Our web collection on statistics for biologists contains articles on many of the points above.

Software and code

Policy information about availability of computer code

Data collection	The contrast enhanced ultrasound (CEUS) and pulsed wave Doppler ultrasound data were acquired using a commercially/clinically available ultrasound system: Siemens ACUSON Sequoia with a 9EC4 transducer (Siemens Medical Solutions, PA). The commercially available contrast agent was Lumason (Bracco Diagnostics Inc., NJ). The cerebral microdialysis data was collected using a commercial, automated ISCUS Flex Microdialysis Analyzer (mDialysis, Sweden) and analyzed using commercially-available LABpilot software (V2.4.0.0, mDialysis, Sweden). The ICP was measured using a commercial invasive ICP monitor (NEUROVENT PTO, Germany). Details are provided in the methods section. The hemodynamic measurements were performed following procedures described in Marquez et al. (Oxygen Exposure During Cardiopulmonary Resuscitation Is Associated With Cerebral Oxidative Injury in a Randomized, Blinded, Controlled, Preclinical Trial. J. Am. Heart Assoc. 9, e015032, 2020).

Data analysis	Details about the analysis procedures of the CEUS data are provided in the methods section. Preprocessing and filtering of the ultrasound images to determine the location of the bubbles (blind-deconvolution, modified histogram equalization) were performed using a custom code written in MATLAB 2018b (Mathworks, Cambridge, MA, USA), which was described in a previous publication (Zhang et al. Time-Resolved Echo-Particle Image/Tracking Velocimetry Measurement of Interactions Between Native Cardiac Output and Veno-Arterial ECMO Flows. J. Biomech. Eng. 143 (2), No. 021008, 2021). Tracking of the bubbles, identification of micro-vessels, removal of large blood vessels, generation of circulation maps, and CMC calculations were performed using a newly developed custom code, also written in MATLAB 2018b. As required, this code is available to the editors and reviewers upon request. Furthermore, the codes will be made available to the scientific community upon request.<br>The time-intensity variations of the bolus injection or disruption-replenish CEUS sequences were calculated using available functions in the Image Processing Toolbox of MATLAB.<br>The statistical analysis and plotting of data were performed using MATLAB functions, as well as Graphpad Prism (V8.3.0, GraphPad Software, USA).<br>The cerebral microdialysis data was analyzed using commercially-available LABpilot software (V2.4.0.0, mDialysis, Sweden).

For manuscripts utilizing custom algorithms or software that are central to the research but not yet described in published literature, software must be made available to editors and reviewers. We strongly encourage code deposition in a community repository (e.g. GitHub). See the Nature Portfolio guidelines for submitting code & software for further information.

Data

Policy information about availability of data

All manuscripts must include a data availability statement. This statement should provide the following information, where applicable:

- Accession codes, unique identifiers, or web links for publicly available datasets
- A description of any restrictions on data availability
- For clinical datasets or third party data, please ensure that the statement adheres to our policy

The data discussed in the paper are available either in the main text or as supplementary material. Source data used in the figures are also provided with this paper. The data in the circulation maps, several GB, are available upon reasonable request to the corresponding author, in accordance with the guidelines of the institutions involved (Johns Hopkins University and Children's Hospital of Philadelphia). The size of the raw CEUS image files exceeds 1 TB, i.e., they are too large to be readily shared online, but could be copied & sent upon reasonable request.

Field-specific reporting

Please select the one below that is the best fit for your research. If you are not sure, read the appropriate sections before making your selection.

☒ Life sciences ☐ Behavioural & social sciences ☐ Ecological, evolutionary & environmental sciences

For a reference copy of the document with all sections, see nature.com/documents/nr-reporting-summary-flat.pdf

Life sciences study design

All studies must disclose on these points even when the disclosure is negative.

Sample size	The present study was the first to measure and map the regional cerebral micro-circulation (CMC) as a function of the intracranial pressure (ICP). Consequently, we could not estimate the mean and standard deviation of the regional cerebral microcirculation (CMC) prior to the experiments, hence we could not estimate the sample size prior to the experiments. The current findings were based on tests involving 8 pigs, each exposed to varying ICP. The primary findings showed a coefficient of determination in the 0.72 to 0.84 range, and correlations of 0.85 to 0.92. Hence, the sample size (n=8), each involving 4-7 ICP levels, appeared to be sufficient for demonstrating the claimed trends.
Data exclusions	All the collected data recorded in the same coronal plane were included in the analysis. However, for one case, the baseline data at the lowest ICP was erroneously acquired in a different plane. Hence, it was not included in the results. We did not exclude any results as outliers.
Replication	As described in the methods section, the present velocity measurements based on bubble tracking were compared to pulsed wave doppler data obtained in the same blood vessel. For the overlapping range, where both velocity measurements were effective, namely 1.0 - 11 cm/s, a Bland-Altman analysis showed that the results agreed well with a standard deviation of 1.2 cm/s. The major findings of this paper, i.e., the functional relationship between cerebral micro-circulation and ICP were consistent and reproducible among the eight pigs.
Randomization	Randomization was not relevant to this study because all the pigs as well as the entire database were treated in the same manner and were not divided into groups.
Blinding	Blinding was not relevant, and data acquisition and analysis were not performed blindly. The conclusions are based on quantitative analysis of measured data, and is unlikely to be affected by observer bias.

Reporting for specific materials, systems and methods

We require information from authors about some types of materials, experimental systems and methods used in many studies. Here, indicate whether each material, system or method listed is relevant to your study. If you are not sure if a list item applies to your research, read the appropriate section before selecting a response.

Materials & experimental systems

n/a	Involved in the study	
☒	☐	Antibodies
☒	☐	Eukaryotic cell lines
☒	☐	Palaeontology and archaeology
☐	☒	Animals and other organisms
☒	☐	Human research participants
☒	☐	Clinical data
☒	☐	Dual use research of concern

Methods

n/a	Involved in the study	
☒	☐	ChIP-seq
☒	☐	Flow cytometry
☒	☐	MRI-based neuroimaging

Animals and other organisms

Policy information about studies involving animals; ARRIVE guidelines recommended for reporting animal research

Laboratory animals	This study involved eight 4-week-old female Yorkshire piglets (9.5~11.9kg).
Wild animals	This study did not involve wild animals.
Field-collected samples	This study did not involve field-collected samples.
Ethics oversight	The current protocol was approved by the Institutional Animal Care and Use Committee of the Children's Hospital of Philadelphia.

Note that full information on the approval of the study protocol must also be provided in the manuscript.
